# Extracts from Argentinian native plants reverse fluconazole resistance in *Candida* species by inhibiting the efflux transporters Mdr1 and Cdr1

**DOI:** 10.1186/s12906-022-03745-4

**Published:** 2022-10-12

**Authors:** Florimar Gil, Jerónimo Laiolo, Brayan Bayona-Pacheco, Richard D. Cannon, Antonio Ferreira-Pereira, María Cecilia Carpinella

**Affiliations:** 1 Fine Chemical and Natural Products Laboratory, IRNASUS CONICET-UCC, Universidad Católica de Córdoba, Avda. Armada Argentina 3555, X5016DHK Córdoba, Argentina; 2 Department of Medicine, Division of Health Science, Universidad del Norte, Km 5, Vía Puerto Colombia, Área Metropolitana de Barranquilla, 081007 Barranquilla, Colombia; 3 Laboratory of Microbial Biochemistry, Institute of Microbiology Paulo de Góes, Universidade Federal Do Rio de Janeiro, Ilha Do Fundão, Av. Carlos Chagas Filho, 373, Bloco I, Sala 44, Rio de Janeiro, RJ 21949-902 Brazil; 4 Faculty of Dentistry, Sir John Walsh Research Institute, University of Otago, PO Box 647, Dunedin, 9054 New Zealand

**Keywords:** Multidrug resistance, Efflux transporter, Mdr1, Cdr1, *Candida albicans*, *Candida glabrata*, *Nakaseomyces glabrata*, ATP binding cassette, Major facilitator superfamily

## Abstract

**Background:**

The development of multidrug resistance (MDR) associated with the overexpression of the efflux transporters Mdr1 and Cdr1 in *Candida* species impedes antifungal therapies. The urgent need for novel agents able to inhibit the function of both pumps, led us to evaluate this property in 137 extracts obtained from Argentinian plants.

**Methods:**

The ability of the extracts to reverse efflux pump-mediated MDR was determined with an agar chemosensitization assay using fluconazole (FCZ) resistant Mdr1- and Cdr1-overexpressing clinical isolates of *Candida albicans* and *Candida glabrata* as well as *Saccharomyces cerevisiae* strains selectively expressing Mdr1 (AD/CaMDR1) or Cdr1 (AD/CaCDR1). The resistance-reversing activity of the most potent extracts was further confirmed using a Nile Red accumulation assay.

**Results:**

Fifteen plant extracts overcame the FCZ resistance of *Candida albicans* 1114, which overexpresses CaMdr1 and CaCdr1, and AD/CaMDR1, with those from *Acalypha communis* and *Solanum atriplicifolium* being the most effective showing 4- to 16-fold reversal of resistance at concentrations ≥ 25 µg/mL. Both extracts, and to a lesser extent that from *Pterocaulon alopecuroides*, also restored FCZ sensitivity in CgCdr1-overexpressing *C. glabrata* 109 and in AD/CaCDR1 with fold reversal values ranging from 4 to 32 and therefore demonstrating a dual effect against Mdr1 and Cdr1. Both, *A. communis* and *S. atriplicifolium* extracts at concentrations ≥ 12.5 and ≥ 25 µg/mL, respectively, increased the intracellular Nile Red accumulation in all yeast strains overexpressing efflux pumps.

**Conclusions:**

The non-toxic and highly active extracts from *A. communis* and *S. atripicifolium*, provide promising sources of compounds for potentiating the antifungal effect of FCZ by blocking the efflux function of Mdr1 and Cdr1 transporters.

**Supplementary Information:**

The online version contains supplementary material available at 10.1186/s12906-022-03745-4.

## Background

Human health has been threatened by microbial infections since antiquity [1]. Among these, infections caused by fungal pathogens have had an enormous impact on public health [2, 3]. Approximately a billion people suffer from mild superficial fungal infections, while more than 150 million people, in particular immunocompromised patients, have severe and life-]. These serious systemic mycoses account for more than a million deaths per annum [threatening invasive diseases caused by fungi [4,5], with a 28–61% mortality rate attributed to candidiasis [6–8]. Although *Candida albicans* remains the main opportunistic fungus causing infections in hospitalized patients, other non-*albicans* species are emerging as prominent pathogens, with *C*. *glabrata* being the most common among these [9, 10]. A crucial factor that contributes to the severity of *Candida* infections is the development of multidrug resistance (MDR), in particul ar to one of the most relevant antifungal class, the triazoles and its first-line agent, fluconazole(FCZ) [11–13]. One of the most important MDR mechanisms leading to azole therapy failure is the overexpression of efflux pumps [12, 14]. These membrane proteins can extrude antifungal drugs from fungi, preventing their intracellular accumulation and thereby rendering these cells insensitive to their therapeutic effect [15]. The most common drug efflux transporters expressed by *C. albicans* are Mdr1 and Cdr1 [14, 16]. The drug/H^+^ antiporter Mdr1, belongs to the major facilitator superfamily (MFS) of proteins [17]. Mdr1 comprises 12 TMHs linked by hydrophilic loops, with the antiporter motif for the translocation of drugs across the membrane, present in TMH5 [18]. Cdr1 belongs to the pleiotropic drug resistance (PDR) subfamily of the ATP binding cassette (ABC) family of transporters, which hydrolyze ATP to provide the energy for the active drug efflux [19]. As an archetype ABC transporter, Cdr1 consists of two transmembrane domains (TMDs) each with six transmembrane helices (TMH) connected by extra and intracellular loops and two nucleotide binding domains (NBDs) [14, 20]. In *C. glabrata*, the *C. albicans* Cdr1 ortholog CgCdr1, is the most prominent efflux pump implicated in azole resistance [21, 22].

Given the important role of drug resistance in the failure of fungal therapy, there is a critical need for alternative antifungals or the development of novel strategies to overcome the MDR phenotype conferred by efflux transporters.

Plants are a great resource for the discovery of compounds with pharmacological potential [23–29], with some plant extracts or extract-derived components belonging to different chemical families, targeting Mdr1 or Cdr1 [30–32]. The immense chemical diversity of secondary metabolites from plants and the low number of species explored to date [33], encourage the scientific community and the pharmaceutical industry to include these products in efflux pump inhibitor (EPI) discovery pipelines.

With the aim of identifying promising reservoirs of novel compounds with the capacity to circumvent MDR by inhibiting *C. albicans* and *C. glabrata* efflux transporters Mdr1 and Cdr1, 137 extracts from plants of Argentina were evaluated.

## Methods

### Materials and reagents

Nile Red and betulin were purchased from Sigma-Aldrich (St. Louis, USA). The antifungal agent FCZ (purity 98.5%) was obtained from Parafarm (Buenos Aires, Argentina). FK506 (tacrolimus, purity ≥ 98%) was purchased from Carbosynth Ltd, UK. Sterile plastic laboratory products were purchased from Greiner Bio-One (Frickenhausen, Germany). All solvents were HPLC grade.

### Plant materials and extract preparation

Aerial parts of plants (listed in Table S1, Supporting Information), were collected from December to March in the hills of Córdoba Province, Argentina, between -30.773428 to -31.797760 latitude and—64.109384 to—64.546803 longitude. Plants were chosen with regards to their availability and the accessibility, and scarce or absence of scientific information concerning their pharmacological and/or phytochemical profiles. Powdered material was extracted by maceration with 96% ethanol (3:1) for 48 h. The yield of each extract obtained after exhaustive solvent removal and expressed as percentage weight of plant material, is depicted in Table S1. Extract solutions were prepared in 96% ethanol just prior to use.

### Strains and culture conditions

A clinical isolate of *C. albicans*, overexpressing CaMdr1 and CaCdr1 (strain 1114) [34] and the CgCdr1-overexpressing *C. glabrata* clinical isolate 109 [35, 36], both obtained from patients at the University Hospital of Universidade Federal de Juiz de Fora, Minas Gerais, were used. The strains were identified by MALDI-TOF mass spectroscopy [35, 36]. Minimum inhibitory concentration (MIC) values, determined with an agar dilution assay as described below, revealed that strain 1114 was 515-times more resistant to FCZ than the sensitive strain *C. albicans* ATCC 90028 (MIC_FCZ_ = 500 and 0.97 µg/mL, respectively) [37], whereas strain 109 displayed 64-fold more resistance to FCZ than *C. glabrata* ATCC 2001 (MIC_FCZ_ = 1,000 and 15.6 µg/mL, respectively) [37]. Both ATCC strains were used in the various assays for comparison purposes. In addition, a set of *Saccharomyces cerevisiae* strains overexpressing specific efflux pumps [38, 39], were included to relate effects to particular pumps. A sensitive null mutant (AD1-8u^−^) in which seven MDR efflux pump genes are deleted [MIC_FCZ_ = 2.0 µg/mL (agar dilution assay – see below)], and its FCZ-resistant derivatives: the AD/CaMDR1 strain that selectively overexpresses *C. albicans* Mdr1 (MIC_FCZ_ = 31.2 µg/mL by agar dilution assay) and the AD/CaCDR1 mutant strain that overexpresses *C. albicans* Cdr1 (MIC_FCZ_ = 250 µg/mL by agar dilution test) were used. Clinical isolates 1114 and 109 were kindly gifted by Dr. Antonio Ferreira-Pereira from the Universidade Federal do Rio de Janeiro while the *S. cerevisiae* strains were kindly gifted by Dr. Richard Cannon from the University of Otago. The mean fluorescence intensity (MFI) of Nile Red retained in untreated cells corresponded to 342, 416, 2,280 and 2,900 MFI units for strains 1114, 109, *C. albicans* ATCC 90028 and *C. glabrata* ATCC 2001, respectively and to 13,700, 4,730 and 52,900 MFI units for strains AD/CaMDR1, AD/CaCDR1 and AD1-8u^−^, respectively. The significantly lower MFI values (*p* < 0.05) for the resistant strains than for the control strains indicated an active efflux of Nile Red from resistant cells.

All yeast strains were stored at -20 °C in Sabouraud dextrose broth (SDB; Difco Laboratories, Detroit, MI, USA) containing 10% glycerol. Prior to use, strains were incubated on Sabouraud agar (Laboratorio Britania, Buenos Aires, Argentina), or in SDB, at 30 °C for 24 h. Cells from Sabouraud agar plates were used to prepare working suspensions in sterile saline.

### Antifungal susceptibility assay

To assess if the extracts exerted antifungal activity per se, an agar dilution test was performed as described previously [28], with some modifications. Solid assays were preferred over liquid methods due to the characteristics of some extracts as they interfered with absorbance readings and/or caused precipitation. In short, molten Sabouraud agar medium was added to duplicate extract solutions to reach a final concentration of 200 µg/mL and poured into wells in 12-well microtiter plates. Suspensions of the target yeasts (2 µl of 6 × 10^5^ cells/mL) were seeded on the surface of the solidified agar, and plates were incubated at 30 °C for 48 h. Negative control wells contained 5% final concentration of ethanol (the highest concentration of ethanol needed to completely solubilize the necessary mass of extract to achieve 200 µg/mL that at the same time did not affect yeast viability). The whole panel of extracts was screened using the four *Candida* strains and those extracts showing reversing properties on Mdr1- and Cdr1-expressing *Candida* strains, as determined by agar chemosensitization assay (see below), were further assayed using the *S. cerevisiae* mutants. The extracts showing a complete inhibition of microorganism growth in the primary screen, were then evaluated at decreasing concentrations to establish their MIC values. The MIC was defined as the minimum concentration of sample that completely inhibited the growth of the yeasts, as determined by visual observation.

To determine the MIC values for FCZ, which was also solubilized in ethanol, final concentrations of 125–2,000 and 0.48–125 µg/mL for the pump-expressing and pump-deficient yeasts, respectively, were used.

At least three separate replicates were performed for each assay.

### Agar chemosensitization assay

The capability of the panel of extracts to sensitize the resistant clinical *Candida* strains to the toxic effect of FCZ, was determined using the antifungal susceptibility protocol except that the extracts at 200 µg/mL or at sub-MICs were added in combination with FCZ at 1/4 of the MIC (125 and 250 µg/mL in the assays with *C. albicans* 1114 and *C. glabrata* 109, respectively). The extracts showing sensitization activity at the initial maximum concentration were further evaluated against all the target strains, including resistant *S. cerevisiae* mutants, with serial dilutions to determine their minimum effective concentration (MEC) with FCZ at varying sub-MICs (1/2 to 1/64 of the MIC).

Betulin, is a triterpene isolated from *Ligaria cuneifolia* that showed potent inhibition of the human ABC transporter, P-glycoprotein (P-gp) at concentrations ≥ 0.39 µM [29]. This property encouraged us to evaluate the capacity of this compound to inhibit Mdr1 and Cdr1. As the concentration of betulin in the 200 µg/mL *L. cuneifolia* (parasitizing *Vachellia* sp.) extract was 8.6 µg/mL (19 µM), betulin was tested at the concentration range 6.25–50 µM.

FCZ sensitive *Candida* spp. and AD1-8u^−^ were simultaneously assayed to determine if there were any synergistic effects other than that involving the transporters (extracts were used at 200 µg/mL and FCZ at 1/4 MIC: 0.24, 3.90 and 0.50 µg/mL in the assays with *C. albicans* 90028, *C. glabrata* 2001 and AD1-8u^−^, respectively). FK506, an inhibitor of Cdr1 [40], was used as a positive control, while ethanol at 5% was used as negative control. Fold reversal (FR) values were calculated as the ratio between the MIC with FCZ alone and the MIC of FCZ in the presence of the extract, from the results obtained in at least three independent experiments.

### Nile Red accumulation assay

The capacity of the most active extracts to cause cells to accumulate the fluorescent cell-associated substrate of Mdr1 and Cdr1, Nile Red [41–43], was monitored by flow cytometry. Briefly, resistant and sensitive yeasts grown to exponential phase in SDB were centrifuged, washed two times with distilled water and kept on ice for 2 h [34]. Then, cells were incubated in 96-well microtitre plates (1 × 10^6^ cells /well) containing SDB in the absence or presence of different concentrations of the extracts dissolved in DMSO at 30 °C for 2 h with shaking at 200 rpm. Then, Nile Red solution was added to each well (7 µM final concentration, with 2% glucose for assays with Cdr1) and cells were incubated at 30 °C for an additional 1.5 h with shaking at 200 rpm. Cells treated only with extracts, to rule out autofluorescence, or with Nile Red and FK506 (20 and 40 µM) as a positive control, were also run. Negative controls contained 1% DMSO instead of plant extract. Then, the fluorescence intensity of 25,000 cells was analyzed with a Life Technologies Attune-NxT flow cytometer with 96-well autosampler (Thermo Fisher Scientific, USA) using a 488 nm excitation laser and a 574/26 nm emission filter. The Nile Red fluorescence signal was analyzed using the Flowjo software (Tree Star, Inc. Ashland, OR). The results were expressed as fluorescence intensity ratio (FIR) values, calculated as the ratio of the MFI of the Nile Red in cells with the addition of the extract to the MFI of the Nile Red in the negative control cells [44, 45]. The MFI values of the negative control cells were comparable to that of Nile Red alone. Mean values were obtained from at least three independent experiments.

### Cytotoxic effect on mammalian cells

To determine the potential cytotoxic effect of the extracts on peripheral blood mononuclear cells (PBMCs), used as a model of normal cells, an MTT assay was performed as previously reported [44, 46]. A hemolysis assay was carried out on red blood cells as described previously [47, 48]. The extracts, dissolved in DMSO, were evaluated at concentrations ranging from 12.5 to 200 µg/mL, DMSO at 1% was used as the negative control. The use of human blood, was approved by the Catholic University of Córdoba Research Ethics Board and all the participants gave written consent.

## Results

### Antifungal activity of extracts

Prior to evaluating the Mdr1/Cdr1 inhibitory activity of the plant extracts, their potential antifungal properties were determined, as the crude extracts likely contained several compounds with different bioactivities, including possible fungitoxic effects.

The screening of the 137 extracts from the plants described in Table S1 showed that they did not inhibit the growth of *C. albicans* 1114, *C. glabrata* 109, *C. albicans* 90028 or *C. glabrata* 2001 at the highest tested concentration (200 µg/mL). Extracts that showed anti-Mdr1 or anti-Mdr1/Cdr1 effect (see below) were further evaluated against the *S. cerevisiae* strains. Only extracts obtained from *Acalypha communis, Argemone subfusiformis, Baccharis salicifolia, Lithrea molleoides* and *Pterocaulon alopecuroides* showed antifungal effects with MIC values of 50, 200, 200, 25 and 100 µg/mL, respectively, against both AD/CaMDR1 and AD1-8u^−^. Therefore, sub-MIC values of these extracts were used in the chemosensitization and Nile Red accumulation assays using AD/CaMDR1 and AD1-8u^−^.

### Chemosensitization of yeast to FCZ

In order to identify plant-derived extracts as sources of efflux pump inhibitors to circumvent MDR in pathogenic yeasts, 137 extracts (Table S1) were investigated. The screen for the ability of these extracts to overcome drug-efflux activity in the resistant *C. albicans* strain 1114, with increased expression of CaMdr1 and CaCdr1, showed that extracts obtained from *A. communis, A. subfusiformis, B. salicifolia, Flourensia campestris, F. oolepis, L. cuneifolia, L. molleoides, Lorentzianthus viscidus, Monnina dictyocarpa, P. alopecuroides*, *Solanum atriplicifolium*, *S. palinacanthum* and *S. salicifolium* improved the antifungal activity of FCZ, with fold-reversal (FR) of FCZ resistance values ranging from 2 to 16 (Table 1). All these extracts also decreased the MIC of FCZ for the resistant yeast AD/CaMDR1. While extracts from *A. communis*, *L. cuneifolia*, *L. viscidus*, *S. atriplicifolium* and *S. salicifolium,* showed similar level of activity against strain 1114 and AD/CaMDR1, the rest of the extracts gave higher FR and/or lower MEC values.

**Table 1:** Reversal of FCZ resistance in yeast cells overexpressing Cdr1 and Mdr1 by extracts from Argentinian flora

Plant species	Extract concentration (µg/mL)	FR			
		*Candida albicans* 1114 (CaMdr1/CaCdr1)	*Saccharomyces cerevisiae* AD/CaMDR1	*Saccharomyces cerevisiae* AD/CaCDR1	*Candida glabrata* 109 (CgCdr1)
*Acalypha communis*	200	16	Nd	32	16
	100	16	Nd	8	16
	50	8	Nd	8	8
	25	4	8	4	8
	12.5	-	-	-	-
*Argemone subsusiformis*	200	2	Nd	-	-
	100	-	16	-	-
	50	-	-	-	-
*Baccharis salicifolia*	200	2	Nd	-	-
	100	-	32	-	-
	50	-	16	-	-
	25	-	4	-	-
	12.5	-	-	-	-
*Flourensia campestris*	200	2	32	-	-
	100	-	32	-	-
	50	-	16	-	-
	25	-	4	-	-
	12.5	-	-	-	-
*Flourensia oolepis*	200	2	16	4	-
	100	-	16	-	-
	50	-	4	-	-
	25	-	-	-	-
*Ligaria cuneifolia* (host *Condalia buxefolia*)	200	2	2	-	-
	100	-	-	-	-
*Ligaria cuneifolia* (host *Lithrea molleoides*)	200	2	2	-	-
	100	-	-	-	-
*Ligaria cuneifolia* (host *Vachelia sp.*)	200	2	2	-	-
	100	-	-	-	-
*Lithrea molleoides*	200	2	Nd	-	-
	100	-	Nd	-	-
	50	-	Nd	-	-
	25	-	Nd	-	-
	12.5	-	2	-	-
	6.25	-	-	-	-
*Lorentzianthus viscidus*	200	2	2	-	-
	100	-	-	-	-
*Monnina dictyocarpa*	200	2	8	-	-
	100	-	4	-	-
	50	-	-	-	-
*Pterocaulon alopecuroides*	200	4	Nd	4	8
	100	-	Nd	-	2
	50	-	8	-	-
	25	-	-	-	-
*Solanum atriplicifolium*	200	8	8	8	8
	100	4	4	4	8
	50	4	-	-	8
	25	4	-	-	4
	12.5	-	-	-	-
*Solanum palinacanthum*	200	2	8	-	-
	100		4	-	-
	50	-	-	-	-
*Solanum salicifolium*	200	2	2	-	-
	100	-	-	-	-
FK506	40 µM	16	Nt	8	16
	20 µM	4	Nt	8	8

*FR* Fold Reversal. -: absence of activity, *Nd* not determined due to the antifungal activity of the extracts at these concentrations, *Nt* not tested since FK506 is a classical inhibitor of ABC efflux pumps [40]

Extracts from *A. communis* and *S. atriplicifolium* displayed the most potent chemosensitizing effect, against both pump-expressing yeasts, with FR values ranging from 4 to 16 and MEC values as low as 25 µg/mL (Table 1). Both extracts, together with that from *P. alopecuroides*, were also effective in restoring FCZ susceptibility in the AD/CaCDR1 strain and in *C. glabrata* 109 that overexpresses CgCdr1 reversing the ABC/MDR resistant phenotype up to 32-fold and showing activity even at 25 µg/mL (Table 1). The rest of the extracts had no effect on the FCZ susceptibility of either AD/CaCDR1 strain or *C. glabrata* 109.

The chemosensitizing efficacy of *A. communis* and of *S. atriplicifolium,* extracts was comparable (*p* > 0.05) to that of the reference compound FK506 (Table 1).

The active extracts did not enhance the antifungal effect of FCZ on the sensitive strains *C. albicans* ATCC 90028*, C. glabrata* ATCC 2001 or *S. cerevisiae* AD1-8u^−^.

On the other hand, betulin at 50 µM did not restore FCZ activity on any of the *Candida* spp. or *S. cerevisiae* strains.

### Nile Red accumulation

In view of the potent FCZ resistance reversal activity displayed by *A. communis* and *S. atriplicifolium* extracts, the ability of these to restore Nile Red accumulation in yeast cells was evaluated.

**Fig. 1:**
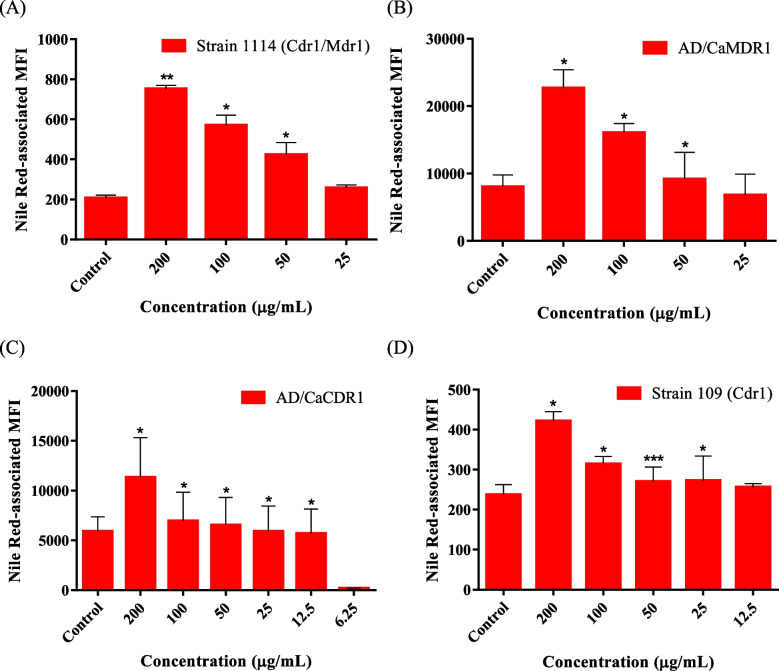
Effects of *Acalypha communis* extract on Nile Red accumulation in: (**A**) *Candida albicans* 1114; (**B**) *Saccharomyces cerevisia*e AD/CaMDR1; (**C**) *S. cerevisia*e AD/CaCDR1; and (**D**) *C. glabrata* 109. The intracellular Nile Red levels increased significantly in the cells treated with different concentrations of the extract. Significant differences from the negative control were determined by using paired one-tailed Student’s *t* test (***p < 0.001, **p < 0.01, *p < 0.05)

**Fig. 2:**
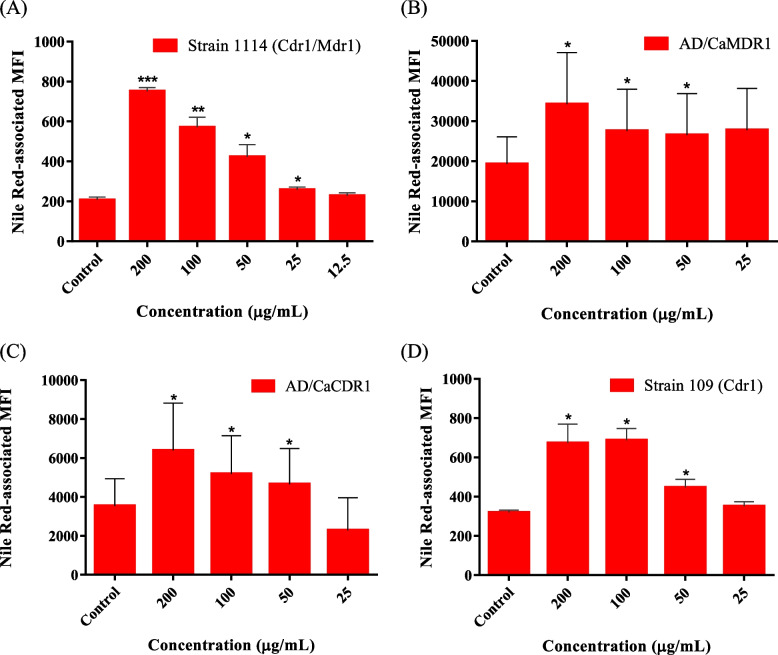
Effects of *Solanum atriplicifolium* extract on Nile Red accumulation in: (**A**) *Candida albicans* 1114; (**B**) *Saccharomyces cerevisiae* AD/CaMDR1; (**C**) *S. cerevisia*e AD/CaCDR1; and (**D**) *C. glabrata* 109. The intracellular Nile Red levels increased significantly in the cells treated with different concentrations of the extract. Significant differences from the negative control were determined by using paired one-tailed Student’s *t* test (***p < 0.001, **p < 0.01, *p < 0.05)

Both extracts strongly inhibited Mdr1- and Cdr1-mediated Nile Red efflux in strains *C. albicans* 1114, AD/CaMDR1, AD/CaCDR1 and *C. glabrata* 109, showing FIR values ranging from 1.54 to 3.68 at concentrations of 200 µg/mL (Table 2). As expected, the extracts showed no effect on Nile Red efflux in both sensitive *Candida* strains and in the null mutant of *S. cerevisiae* (Table 2). *A. communis* and *S. atriplicifolium* extracts were still able to enhance Nile Red retention (*p* < 0.05) at 50 and 25 µg/mL, respectively, in *C. albicans* 1114; at 50 µg/mL in AD/CaMDR1; at 12.5 and 50 µg/mL, respectively, in AD/CaCDR1; and at 25 and 50 µg/mL, respectively, in *C. glabrata* 109 (Table 2 and Figs. 1 and 2).

**Table 2:** Inhibitory effect of extracts from *Acalypha communis* and *Solanum atripicifolium* on Nile Red transport in yeast cells overexpressing Mdr1 and Cdr1 transporters

Yeast	Plant species	FIR					
		Extract concentration (µg/mL)					
		200	100	50	25	12.5	6.25
*Candida albicans* 1114 (CaMdr1/CaCdr1)	*Acalypha communis*	2.36 ± 0.41^**^	1.69 ± 0.07^**^	1.24 ± 0.17^*^	1.07 ± 0.12		
	*Solanum atripicifolium*	3.68 ± 0.36^***^	2.82 ± 0.45^**^	2.11 ± 0.46^*^	1.27 ± 0.17^*^	1.12 ± 0.15	
*Candida albicans* ATCC 90028	*Acalypha communis*	0.30 ± 0.02^**^					
	*Solanum atripicifolium*	1.12 ± 0.19					
*Saccharomyces cerevisiae* AD/CaMDR1	*Acalypha communis*	1.92 ± 0.23^*^	1.74 ± 0.07^***^	1.53 ± 0.10^*^	1.17 ± 0.16		
	*Solanum atripicifolium*	1.93 ± 0.16^*^	1.64 ± 0.19^*^	1.26 ± 0.05^*^	1.11 ± 0.07		
*Saccharomyces cerevisiae* AD/CaCDR1	*Acalypha communis*	2.10 ± 0.13^*^	1.94 ± 0.25^*^	1.52 ± 0.18^*^	1.25 ± 0.15^*^	1.10 ± 0.14^*^	0.80 ± 0.08
	*Solanum atripicifolium*	2.01 ± 0.12^*^	1.68 ± 0.14^*^	1.34 ± 0.06^*^	1.07 ± 0.07		
*Saccharomyces cerevisiae* AD1-8u^−^	*Acalypha communis*	0.70 ± 0.05^*^					
	*Solanum atripicifolium*	0.81 ± 0.09					
*Candida glabrata* 109 (CgCdr1)	*Acalypha communis*	1.54 ± 0.18^*^	1.35 ± 0.10^*^	1.23 ± 0.12^***^	1.26 ± 0.17^*^	1.16 ± 0.11	
	*Solanum atripicifolium*	2.11 ± 0.31^*^	2.15 ± 0.19^*^	1.39 ± 0.10^*^	1.10 ± 0.08		
*Candida glabrata* ATCC 2001	*Acalypha communis*	0.30 ± 0.04					
	*Solanum atripicifolium*	0.62 ± 0.04					

*FIR* Fluorescence intensity ratio, *MFI* mean fluorescence intensity of Nile Red in cells incubated with extract/MFI of Nile Red in cells incubated without extract. Significant differences with respect to the negative control were determined by using a paired one-tailed Student’s *t* test (^***^p < 0.001, ^**^p < 0.01, ^*^p < 0.05)

The Nile Red accumulation in assays performed with *A. communis* at 200 µg/mL in *C. albicans* 1114 and AD/CaCDR1 and with the same concentration of *S. atriplicifolium* in AD/CaCDR1 and *C. glabrata* 109 was similar (*p* > 0.05) to that in cells treated with FK506 at 40 µM (FIR values of 2.05 ± 0.06^***^, 1.80 ± 0.36^**^, 2.68 ± 0.06^**^ against strains 1114, AD/CaCDR1 and 109, respectively). On the contrary, the accumulation of the dye was significantly lower (*p* < 0.01) in *C. glabrata* 109 treated with *A. communis* extract evaluated at 200 µg/mL and higher (*p* < 0.01) in *C. albicans* 1114 treated with *S. atriplicifolium* extract at the same concentration, respect to yeasts treated with FK506 tested at 40 µM. At the MEC values, both extracts were as active (*p* > 0.05) as FK506 at 20 µM (FIR = 1.46 ± 0.12^*^, 1.49 ± 0.12^***^, 1.58 ± 0.18^*^ against strains 1114, AD/CaCDR1 and 109, respectively) in the strains evaluated, except for *A. communis* and *S. atriplicifolium* extracts assayed in AD/CaCDR1 and *C. glabrata* 109, respectively (*p* < 0.05).

It is important to note that the plant extracts did not exhibit auto-fluorescence.

### Cytotoxic effect on mammalian cells

The toxic effect of the most active extracts on mammalian cells was investigated with MTT and hemolysis assays using PBMC and erythrocytes, respectively. The *A. communis* extract showed a half-maximal inhibitory concentration (IC_50_) value of 64.67 ± 0.99 µg/mL against PBMC and only showed hemolysis at > 100 µg/mL while the *S. atripicifolium* extract exhibited an IC_50_ value of 119.80 ± 0.95 µg/mL and no hemolysis at 200 µg/mL.

## Discussion

In order to discover novel sources of compounds able to counteract the MDR linked to fungal ABC and MFS efflux pumps, a panel of 137 extracts from mostly native plants from Argentina, was screened.

The results indicated that 15 extracts rendered strains *C. albicans* 1114 and AD/CaMDR1 sensitive to FCZ by inhibiting the efflux linked to Mdr1 (Table 1). Among the effective extracts, those obtained from *A. communis* and *S. atriplicifolium* had the greatest ability to chemosensitize strains to FCZ and, together with the *P. alopecuroides* extract, also increased the antifungal effect of the azole in the Cdr1-overexpressing cells, AD/CaCDR1 and *C. glabrata* 109 (Table 1). According to these findings, *A. communis*, *P. alopecuroides* and *S*. *atriplicifolium* extracts behaved as dual inhibitors. The remaining extracts did not reverse the resistance to FCZ in the Cdr1-expressing yeasts, demonstrating a selective activity towards CaMdr1. These results would explain the lower chemosensitization observed for the clinical isolate *C. albicans* 1114 compared to *S. cerevisiae* AD/CaMDR1 for *A. subfusiformis*, *B. salicifolia*, *F. campestris*, *F. oolepis*, *L. molleoides*, *M. dictyocarpa* and *S. palinacanthum* extracts (Table 1), since although the MFS transporter Mdr1 may be targeted in, the ABC pump would be still active in *C. albicans* 1114, thus decreasing the intracellular concentration of FCZ and therefore evading partially its antifungal action.

The low MECs (25 µg/mL) that caused a reduction in the MIC_FCZ_ values of at least fourfold with the clinical isolates of *Candida*, highlighted *A. communis* and *S. atriplicifolium* as promising sources of Mdr1 and Cdr1 inhibitors to augment FCZ activity in the treatment for azole resistant candidiasis. None of the 15 active extracts increased the antifungal effect of FCZ in the sensitive *C. albicans*, *C. glabrata* and AD1-8u^−^ strains, supporting the specific interference with the efflux function of the transporters.

*L. cuneifolia* is a hemiparasitic plant that grows in different hosts [49]. The activity of extracts obtained from this species from each of the host trees, *L. molleoides, Vachellia* sp. and *Condalia buxifolia* was similar (Table 1), suggesting that the parasitized plant did not influence the pump inhibitory effect.

Betulin, isolated from *L. cuneifolia*, has been found to strongly inhibit the human ABC transporter P-gp at micromolar concentrations by blocking the efflux of doxorubicin and consequently restoring the sensitivity of leukemia cells to its cytotoxic effect [29]. However, this triterpene did not reduce FCZ resistance in the target yeasts, even at the high concentration of 50 µM. This result suggests that betulin is a selective inhibitor of P-gp, at least with respect to Mdr1 and Cdr1. Although this result correlated with the lack of activity of *L. cuneifolia* extract against Cdr1, it is not in accordance with the effect of this plant against Mdr1, suggesting that another metabolite with Mdr1 blocking activity is present. The inability of betulin to inhibit the transport of FCZ mediated by yeast efflux pumps concurs with the observation that diterpenoid esters isolated from *Euphorbia* spp. were effective at inhibiting P-gp but were not active against *C. albicans* Mdr1 or Cdr1 [50].

Both *A. communis* and *S. atriplicifolium* extracts inhibited Nile Red transport at concentrations ≥ 12.5 µg/mL (Table 2 and Figs. 1 and 2) with FIR values ranging from 1.10 to 3.68 (Table 2). These results confirmed that the enhanced activity of FCZ was due to an increased intracellular accumulation. In these experiments, the Nile Red MFI values were lower for the untreated and treated *Candida* spp*.* than for the *S. cerevisiae* strains (Figs. 1 and 2). At the emission and excitation wavelengths used in this study (488 nm and a 574/26 nm, respectively), Nile Red labels neutral lipids within intracellular lipid droplets [51, 52]. The differences in the MFI values obtained, suggest that *S. cerevisiae* strains contain more neutral lipids than *Candida* strains.

Although the significantly lower MFI values (*p* < 0.05) for untreated AD/CaCDR1 (4,730) and AD/CaMDR1 (13,700) in comparison to AD1-8u^−^ (MFI = 52,900), showed the effectiveness of the pumps at transporting Nile Red, the lower MFI value for AD/CaCDR1 than for AD/CaMDR1 (*p* < 0.05) indicated that Nile Red is more efficiently effluxed by CaCdr1 than by CaMdr1. Therefore, it is encouraging that *A. communis*, *P. alopecuroides* and *S. atripicifolium* extracts were able to inhibit this proficient ABC-type pump.

The same Nile Red-derived fluorescence in pump-deficient *C. albicans*, *C. glabrata* and AD1-8u^−^ cells in the absence or presence of the extracts suggests that the increased FCZ susceptibility in MDR cells is likely achieved by inhibiting Mdr1 and Cdr1.

As far as we are aware, there have been few studies that have screened libraries of plant extracts for MDR reversal activity through interference with yeast transporters [32, 50]. In particular, no information was found in the literature with respect to this property in *A. communis* and *S. atripicifolium* extracts. The cycloartane triterpenes 16α-hydroxymollic acid, 15α-hydroxymollic acid, and 7β,16β-dihydroxy-1,23-dideoxyjessic acid have been isolated from *A. communis* [53], however there is no evidence that any of the compounds are Cdr1 and/or Mdr1 inhibitors. As far as we are aware, no compounds from *S. atriplicifolium* extracts have been identified. It is important to note that assays were performed with crude plant extracts likely to contain a mixture of compounds. The next step in this research will be to purify the active component(s) from the extracts. These compounds are likely to have a higher pump-inhibitory specific activity.

The *A. communis* and S*. atripicifolium* extracts were devoid of toxic effects on mammalian cells. The IC_50_ values obtained towards PBMC were higher than 20 µg/mL, which is the threshold established by the US National Cancer Institute (NCI) to consider an extract as cytotoxic [26]. In addition, neither extract caused hemolysis of human erythrocytes at concentrations of 100 µg/mL.

## Conclusion

The increasing incidence of MDR in *Candida* species due to the overexpression of efflux pumps, led us to search new sources of compounds with the capacity to circumvent this worldwide problem. We found that 15 extracts, in particular those from *A. communis* and *S. atriplicifolium*, were able to reverse the resistance to FCZ by interfering with the efflux function of the MFS transporter Mdr1 and of the ABC pump, Cdr1. In the case of *A. communis* and *S. atriplicifolium* extracts, this property was observed at a concentration ≥ 25 µg/mL and decreased the resistance to FCZ up to 32-fold. Likewise, these extracts inhibited the efflux of Nile Red mediated by both Mdr1 and Cdr1. The modulation of efflux was not only observed with *S. cerevisiae* strains overexpressing Mdr1 or Cdr1 but also in MDR clinical *Candida* spp. strains, which highlights the therapeutic potential of the extract components. The traditional use of *A. communis* is as a purgative and for the treatment of skin wounds [27]. Although this study could not explain the above mentioned popular uses, it identifies a new biological activity of this plant species. No popular uses have been reported for S. *atriplicifolium*. The promising dual efflux pump inhibitory effect of *A. communis* and *S. atriplicifolium* extracts together with their low toxicity, highlight these as candidates to obtain active principles that can counteract antifungal resistance in *Candida* infections.

## Supplementary Information

**Additional file 1:** **Table S1.** Plants from central Argentina screened for Mdr1 and Cdr1 inhibitory effect. **Fig. S1** Flow cytometric determinations of the effect of *Acalypha communis* extract on Nile Red accumulation in: (A) *Candida albicans *1114; (B) *Saccharomyces cerevisiae* AD/CaMDR1; (C) *Saccharomyces cerevisiae* AD/CaCDR1; and (D) *Candida glabrata* 109. **Fig. S2** Flow cytometric determinations of the effect of *Solanum atriplicifolium* extract on Nile Red accumulation in: (A) *Candida albicans* 1114; (B) *Saccharomyces cerevisiae* AD/CaMDR1; (C) *Saccharomyces cerevisiae* AD/CaCDR1; and (D) *Candida glabrata* 109. **Fig. S3** Determination by flow cytometry of the effect of (A) *Acalypha communis* and (B) *Solanum atriplicifolium* extracts on *Saccharomyces cerevisiae* AD1-8u^-^, *Candida albicans* ATCC 90028 and *Candida glabrata* ATCC 2001. Histograms represent cells treated with Nile Red alone in red and cells treated with Nile Red and extracts in blue.

## Data Availability

The datasets used and/or analysed during the current study are available from the corresponding author on reasonable request. Experimental research and field studies on plants (either cultivated or wild), including the collection of plant material, comply with relevant institutional, national, and international guidelines and legislation.
