# Supplementary material for: Extracts from Argentinian native plants reverse fluconazole resistance in Candida species by inhibiting the efflux transporters Mdr1 and Cdr1
Source: BMC Complement Med Ther. 2022 Oct 12;22:264. doi: 10.1186/s12906-022-03745-4 (PMC9555179; doi:10.1186/s12906-022-03745-4)
Supplement: Supplementary file 1 — Additional file 1: Table S1. Plants from central Argentina screened for Mdr1 and Cdr1 inhibitory effect. Fig. S1 Flow cytometric determinations of the effect of Acalypha communis extract on Nile Red accumulation in: (A) Candida albicans 1114; (B) Saccharomyces cerevisiae AD/CaMDR1; (C) Saccharomyces cerevisiae AD/CaCDR1; and (D) Candida glabrata 109. Fig. S2 Flow cytometric determinations of the effect of Solanum atriplicifolium extract on Nile Red accumulation in: (A) Candida albicans 1114; (B) Saccharomyces cerevisiae AD/CaMDR1; (C) Saccharomyces cerevisiae AD/CaCDR1; and (D) Candida glabrata 109. Fig. S3 Determination by flow cytometry of the effect of (A) Acalypha communis and (B) Solanum atriplicifolium extracts on Saccharomyces cerevisiae AD1-8u-, Candida albicans ATCC 90028 and Candida glabrata ATCC 2001. Histograms represent cells treated with Nile Red alone in red and cells treated with Nile Red and extracts in blue. [file 12906_2022_3745_MOESM1_ESM.docx]

Extracts from Argentinian native plants reverse fluconazole resistance in *Candida* species by inhibiting the efflux transporters Mdr1 and Cdr1

Florimar Gil^1^, Jerónimo Laiolo^1^, Brayan Bayona-Pacheco ^2,3^, Richard D. Cannon^4^, Antonio Ferreira-Pereira^3^ and María Cecilia Carpinella^1,*^

^1^ Fine Chemical and Natural Products Laboratory, IRNASUS CONICET-UCC, Universidad Católica de Córdoba, Avda. Armada Argentina 3555, Córdoba X5016DHK, Argentina.

^2^ Department of Medicine, Division of Health Science, Universidad del Norte, Km 5, vía Puerto Colombia, Área Metropolitana de Barranquilla 081007, Colombia.

^3^ Laboratory of Microbial Biochemistry, Institute of Microbiology Paulo de Góes, Universidade Federal do Rio de Janeiro, Av. Carlos Chagas Filho, 373, Bloco I, Sala 44, Ilha do Fundão, 21949-902 Rio de Janeiro, RJ, Brazil.

^4^ Sir John Walsh Research Institute, University of Otago, Faculty of Dentistry, PO Box 647, Dunedin 9054, New Zealand.

**Table S1.**

Plants from central Argentina screened for Mdr1 and Cdr1 inhibitory effect

| **Plant species** | **Family** | **Statusª** | **Common name** | **Yield (%)** | **Voucher UCCOR number** |
| --- | --- | --- | --- | --- | --- |
|  |  |  |  |  |  |
| *Acalypha communis* Müll. Arg. | Euphorbiaceae | N | acalifa | 11.3 | 190 |
| *Acanthostyles buniifolius* (Hook. & Arn.) R.M. King & H. Rob.) | Asteraceae | N | romerito | 5.2 | 136 |
| *Achyrocline satureioides* (Lam.) DC. | Asteraceae | N | marcela hembra | 4.5 | 140 |
| *Achyrocline tomentosa* Rusby | Asteraceae | N | marcela | 4.3 | 160 |
| *Adesmia muricata* (Jacq.) DC. var. *dentata* (Lag.) Benth | Fabaceae | N |  | 4.6 | 438 |
| *Aldama tucumanensis* (Hook. & Arn.) E.E.Schill. & Panero | Asteraceae | N | sesanquillo | 13.6 | 186 |
| *Aloysia citrodora*  Palau | Verbenaceae | N | cedrón | - | 262 |
| *Aloysia gratissima* (Gill. & Hook.) Tronc. | Verbenaceae | N | palo amarillo | 1.7 | 3 |
| *Alternanthera pungens* Kunth | Amaranthaceae | N |  | 5.0 | - |
| *Ambrosia elatior* L. | Asteraceae | N | altamisa | 2.2 | 215 |
| *Amelichloa brachychaeta* (Godr.) Arraya & Barkwork | Poaceae | N |  | 5.5 | 454 |
| *Amphilophium carolinae* (Lindl.) L.G. Lohmann | Bignonaceae | N | peine de mono | 7.4 | 121 |
| *Anemia tomentosa* (Savigny) Sw. | Schizaceae | N | doradilla | 1.9 | 228 |
| *Anthemis cotula* L. | Asteraceae | Adv | manzanilla cimarrona | 8.0 | 128 |
| *Araujia brachystephana (*Griseb.) Fontella & Goyder | Asclepiadaceae | N | doca, tasi | 5.5 | 155 |
| *Argemone subfusiformis* G. B. Ownbey | Papaveraceae | N | cardo santo | 14.4 | 130 |
| *Aristolochia argentina* Griseb. | Aristolochiaceae | N | charrúa | 6.3 | 191 |
| *Artemisia annua* L. | Asteraceae | Adv | artemisia | 4.6 | 363 |
| *Artemisia verlotorum* Lamotte | Asteraceae | Adv | yuyo de San Vicente | 3.5 | 230 |
| *Aspidosperma quebracho-blanco* Schltdl. | Apocynaceae | N | quebracho blanco | - | 116 |
| *Atriplex cordobensis* Gand. & Stuck. | Chenopodiaceae | N | Atriplex macho | 3.9 | 480 |
| *Atriplex cordobensis* Gand. & Stuck. | Chenopodiaceae | N | Atriplex hembra | 8.2 | 481 |
| *Atriplex nummularia* Lindl. | Chenopodiaceae | Cult. |  | 12.8 | 478 |
| *Baccharis aliena* (Spreng.) Joch.Müll. | Asteraceae | E | romerillo | 23.6 | 194 |
| *Baccharis artemisioides* Hook. & Arn. | Asteraceae | E | romerillo blanco | 2.8 | 142 |
| *Baccharis articulata* (Lam.) Pers | Asteraceae | N | carquejilla | 9.4 | 503-504 |
| *Baccharis cordobensis* Heering | Asteraceae | E |  | 25.2 | - |
| *Baccharis flabellata* Hook. & Arn. | Asteraceae | E | chilquilla | 7.5 | 137 |
| *Baccharis* spp. | Asteraceae | N |  | - | 460 |
| *Baccharis rufescens* Spreng*.* | Asteraceae | E | pichi de la sierra | 4.0 | 261 |
| *Baccharis salicifolia* (Ruiz et Pav.) Pers. | Asteraceae | N | suncho | 9.6 | 175 |
| *Bidens pilosa* L. | Asteraceae | N | amor seco | 4.8 | 213 |
| *Buddleja mendozensis* Griseb. | Buddlejaceae | N | salvialora | 23.5 | 271 |
| *Buddleja cordobensis* Griseb. | Buddlejaceae | E |  | 6.3 | 196 |
| *Calceolaria parviflora* Benth. | Calceolariaceae | E | zapatito | 6.1 | 451 |
| *Cantinoa mutabilis* (Rich.) Harley & J.F.B. Pastore | Lamiaceae | N | verbena negra | 2.8 | 260 |
| *Capparis atamisquea* Kuntze | Capparaceae | N | atamisqui, matagusanos | 3.15 | 188 |
| *Chromolaena hookeriana* (Gris.) R. M. King & H. Rob. | Asteraceae | N |  | 3.4 | 158 |
| *Collaea argentina* Griseb. | Fabaceae | N |  | - | 399 |
| *Condalia microphylla* Cav. | Rhamnaceae | E | piquillín | 1.6 | 180 |
| *Cotoneaster glaucophyllus* Franch. | Rosaceae | I | crategus | 9.1 | 126 |
| *Croton lachnostachyus* Baill. | Euphorbiaceae | N | botonillo | 3.5 | 187 |
| *Croton* sp. | Euphorbiaceae |  |  | - | 448 |
| *Cynoglossum amabile* Stapf & J.R. Drumm. | Boraginaceae | Adv |  | 2.7 | 207 |
| *Dalea elegans* Hook. & Arn. | Fabaceae | E |  | 11.8 | 254 |
| *Dimerostemma aspilioides* (Griseb.) M.D. Moraes | Asteraceae | E |  | 5.5 | 246 |
| *Dipsacus fullonum* L. | Dipsacaceae | I | carda | 5.2 | 124 |
| *Dolichandra cynanchoides* Cham. | Bignonaceae | N | sacha huasca | 4.3 | 120 |
| *Dolichandra unguis-cati* (L.) L.G. Lohmann | Bignonaceae | N | uña de gato (bignonia amarilla) | 9.9 | 265 |
| *Dysphania ambrosioides* (L.) Mosyakin & Clemants | Chenopodiaceae | N | paico | 3.0 | 331 |
| *Echium plantagineum* L. | Boraginaceae | Adv |  | 4.8 | 452 |
| *Elaphoglosum lorentzii* (Hieron.) H. Christ | Lomariopsidaceae | N |  | 10.5 | 234 |
| *Eragrostis curvula* (Schrad.) Nees | Poaceae | I | pasto llorón | 1.39 | 492 |
| *Eryngium horridum* Malme | Apiaceae | N | turututú | 4.7 | 192 |
| *Escallonia cordobensis* (Kuntze) Hosseus | Escalloniaceae | E |  | - | 459 |
| *Flourensia campestris* Griseb. | Asteraceae | E | chilca | 11.8 | 221 |
| *Flourensia oolepis* S.F. Blake | Asteraceae | E | chilca | 23.0 | 135 |
| *Gaillardia megapotamica* (Spreng.) Baker | Asteraceae | E | topasaire | 13.76 | 127 |
| *Gomphrena pulchella* Mart. | Amaranthaceae | N | siempreviva | 3.9 | 403 |
| *Grindelia pulchella* Dunal | Asteraceae | E | botoncito | 6.7 | 205 |
| *Handroanthus heptaphyllus* (Vell.) Mattos | Bignoniaceae | N |  | 2.6 | 502 |
| *Heliotropium amplexicaule* Vahl | Boraginaceae | N | borraja | 2.42 | 531 |
| *Ipomoea hieronymi* (Kuntze) O´Donell | Convolvulaceae | N |  | - | 391 |
| *Jarava ichu* Ruiz et Pav. | Poaceae | N | paja viscachera | 1.17 | 224 |
| *Jasminum mesnyi* Hance | Oleaceae | Adv | jazmín amarillo | 3.1 | 533 |
| *Jodina rhombifolia* (Hook. & Arn*.*) Reissek | Santalaceae | N | sombra de toro | 5.0 | 153 |
| *Lantana balansae* Briq. | Verbenaceae | N |  | 6.2 | 431 |
| *Lantana grisebachii* Seckt. | Verbenaceae | N | lantana de la sierra | 7.15 | 200 y 379 |
| *Lathyrus macropus* Hook. & Arn. | Fabaceae | E | arvejilla | 5.2 | 286 |
| *Lepechinia floribunda* (Benth.) Epling | Lamiaceae | N | salvia blanca | 3.7 | 195 |
| *Lepechinia meyenii* (Walp.) Epling | Lamiaceae | N |  | 3.8 | 233 |
| *Lessingianthus mollissimus* (Hook. & Arn.) H. Rob. | Asteraceae | E |  | 2.0 | 204 |
| *Ligaria cuneifolia* (Ruiz & Pav.) Tiegh.  (parasitizing *Lithrea molleoides*) | Loranthaceae | N | liga | 4.4 | 517 |
| *Ligaria cuneifolia* (Ruiz & Pav.) Tiegh.  (parasitizing *Vachellia* sp.) | Loranthaceae | N | liga | 4.4 | 518 |
| *Ligaria cuneifolia* (Ruiz & Pav.) Tiegh.  (parasitizing *Condalia buxifolia*) | Loranthaceae | N | liga | 9.11 | 522 |
| *Ligustrum lucidum* W. T. Aiton | Oleaceae | Adv | siempreverde | 9.0 | 532 |
| *Lithrea molleoides* (Vell.) Engl. | Anacardiaceae | N | molle | 10.9 | 183 |
| *Lorentzianthus viscidus* (Hook. & Arn.) R.M. King & H. Rob. | Asteraceae | N |  | 2.9 | 139 |
| *Mandevilla laxa* (Ruiz & Pav.) Woodson | Apocynaceae | N | jazmín chileno | 2.4 | 157 |
| *Mandevilla pentlandiana* (A. DC.) Woodson | Apocynaceae | N |  | 10.9 | 189 |
| *Melinis repens* (Wild.) Zizka | Poaceae | I |  | 1.2 | 227 |
| *Melissa officinalis* L. | Lamiaceae | Adv | melisa | 1.0 | 240 |
| *Microliabum candidum* (Griseb.) H. Rob. | Asteraceae | E | salvia de la puna | 3.7 | 220 |
| *Minthostachys verticillata* (Griseb.) Epling | Lamiaceae | E | peperina | - | 125 |
| *Monnina dictyocarpa* Griseb. | Polygalaceae | E | quelen | 8.1 | 277 |
| *Nierembergia linariaefolia* Graham | Solanaceae | E | chuscho | 16.06 | 523 |
| *Nothoscordum gracile* (Dryand. *ex* Aiton) Stearn | Liliaceae | N | lágrimas de la virgen | - | 530 |
| *Ophryosporus charua* (Griseb.) Hieron. | Asteraceae | E |  | 13.6 | 226 |
| *Oplismenus hirtellus* (L.) P. Beauv. ssp. *hirtellus* | Poaceae | N |  | 3.8 | 444 |
| *Otholobium higuerilla* (Gillies ex Hook.) J. W. Grimes | Fabaceae | N | higuerilla | 1.8 | - |
| *Pascalia glauca* Ortega | Asteraceae | N | sunchillo, asolador | 6.4 | 185 |
| *Pavonia aurigloba* Krapov. & Cristóbal | Malvaceae | E |  | 2.3 | 159 |
| *Petunia axillaris* Briton, Stern & Poggenb. | Solanaceae | N |  | 6.0 | 526 |
| *Phacelia pinnatifida* Griseb. *ex* Wedd. | Boraginaceae | N |  | - | 446 |
|  |  |  |  |  |  |
| *Plantago australis* subsp*. australis* Lam. | Plantaginaceae | N |  | 6.8 | 494 |
| *Plantago major* L. | Plantaginaceae | Adv. |  | 6.0 | 495 |
| *Podranea ricasoliana* (Tanfani) Sprague | Bignonaceae | Adv | trompeta rosada | 4.5 | 164 |
| *Polystichum montevidense* (Spreng.) Rosenst. | Dryopteridaceae | N |  | 3.6 | 450 |
| *Porlieria microphylla* (Baill.) Descole, O´Donell & Lourteig | Zygophyllaceae | N | cucharero | 1.1 | 154 |
| *Prosopis* sp*.* | Fabaceae | N | algarrobo | 8.5 | 256 |
| *Pterocaulon alopecuroides* (Lam.) DC. | Asteraceae | N | caraí casó | 5.5 | 217 |
| *Pyrostegia venusta* (Ker Gawl.) Miers | Bignonaceae | N | lluvia de fuego, flor de San Juan | 0.9 | 165 |
| *Rhynchosia diversifolia* Micheli | Fabaceae | N |  | 4.1 | 394 |
| *Salpichroa origanifolia* (Lam.) Baill. | Solanaceae | N | uvita del campo | 3.25 | 529 |
| *Salvia cuspidata* Ruiz & Pav. | Lamiaceae | N | salvia morada | 8.7 | 266 |
| *Schinopsis lorentzii* (Griseb.) Engl. | Anacardiaceae | N | Horco quebracho | - | - |
| *Schizachyrium condensatum* (Kunth) Nees | Poaceae | N | paja colorada | 1.9 | 225 |
| *Schkuhria pinnata* (Lam.) Kuntze ex Thell. | Asteraceae | N | matapulgas | 13.0 | 374 |
| *Senecio madagascariensis* Poir. | Asteraceae | Adv |  | 4.9 | 208 |
| *Senecio viravira* Hieron | Asteraceae | N | viravira | 3.9 | 181 |
| *Senna aphylla* (Cav.) H.S. Irwin et Barneby | Fabaceae | N | pichana, pichanilla, cabello de indio | 5.1 | 174 |
| *Sida rhombifolia* L. | Malvaceae | N | escoba dura | 2.2 | 141 |
| *Solanum argentinum* Bitter & Lillo | Solanaceae | N | duraznillo blanco | 5.4 | 34 |
| *Solanum atriplicifolium* Gillies ex Nees | Solanaceae | N |  | 18.08 | 528 |
| *Solanum palinacanthum* Dunal | Solanaceae | N | pocote | 3.1 | 198 |
| *Solanum salicifolium* Phil. | Solanaceae | E |  | 2.85 | 527 |
| *Solanum sisymbriifolium* Lam. | Solanaceae | N | espina colorada | 2.5 | 199 |
| *Sphaeralcea cordobensis* Krapov. | Malvaceae | E | malvavisco | 3.1 | 177 |
| *Sphaeralceae cordobensis* (mutant) | Malvaceae | E | malva blanca | 1.9 | 182 |
| *Tagetes filifolia* Lagasca | Asteraceae | N |  | 3.4 | 214 |
| *Tagetes argentina* Cabrera | Asteraceae | E |  | 4.3 | 493 |
| *Tagetes campanulata* Griseb. | Asteraceae | N |  | - | 7263 (CORD) |
| *Tagetes minuta* L. | Astereaceae | N | suico | 2.5 | 138 |
| *Thalictrum decipiens* Boivin | Ranunculaceae | N | albaquilla | 4.7 | 229 y 287 |
| *Thelesperma megapotamicum* (Spreng.) Kuntze | Asteraceae | N | té pampa | 5.1 | 303 |
| *Tillandsia capillaris* Ruiz et Pav. | Bromeliaceae | N | clavel del aire | 2.9 | 237 |
| *Tipuana tipu* (Benth.) Kuntze | Fabaceae | N | tipa | - | 479 |
| *Tradescantia fluminensis* Vell. | Commelinaceae | N |  | 4.3 | 443 |
| *Trichocline reptans* (Wedd.) Hieron. | Asteraceae | N | árnica, contrayerba | 6.1 | 244 |
| *Tripodanthus flagellaris* (Cham. & Schltdl.) Tiegh | Loranthaceae | E | liga blanca | 7.2 | 144 |
| *Vachellia aroma* (Gillies *ex* Hook. & Arn.) Seigler & Ebinger | Fabaceae | N | tusca | 29.6 | 242 |
| *Vachellia cavens* (Molina) Seigler & Ebinger | Fabaceae | N | espinillo | 25.2 | - |
| *Verbesina encelioides* (Cav.) Benth. & Hook. *f. ex* A. Gray | Asteraceae | N |  | 4.9 | 364 |
| *Vernonanthura nudiflora (Less.) H. Rob.* | Asteraceae | N |  | - | 129 |
| *Vicia graminea* Sm. | Fabaceae | N | arvejilla silvestre | 4.5 | 285 |
| *Wedelia buphtalmiflora* Lorentz | Asteraceae | E |  | 1.8 | 210 |
| *Zanthoxylum coco* Hook. F. & Arn. | Rutaceae | N | coco, cochucho | 1.7 | 263 |

**^a^**Adv.: Adventive; Cult: Cultivated; E: Endemic; I: Introduced; N: Native.

Nile Red Fluorescence

Nile Red Fluorescence

**Fig. S1**

Nile Red Fluorescence


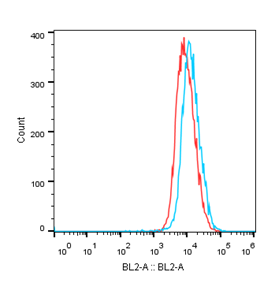

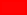


Nile Red

Nile Red + extract 50 µM


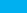

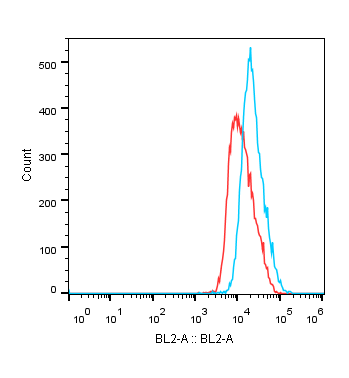


Nile Red Fluorescence


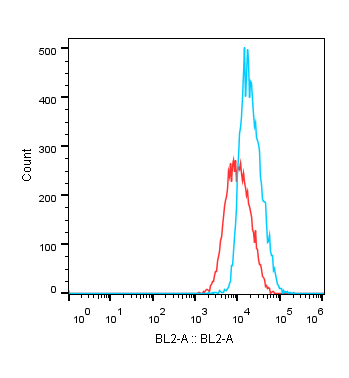

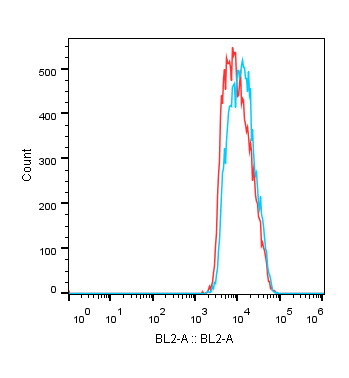

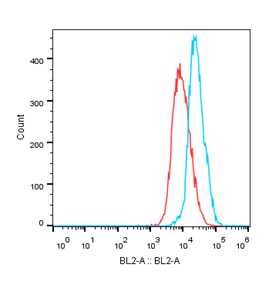


A


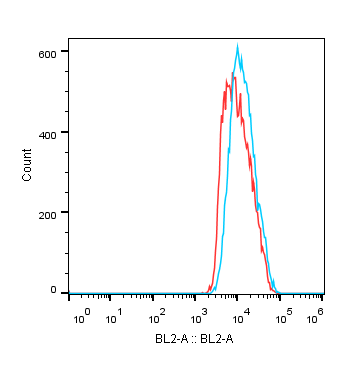


D


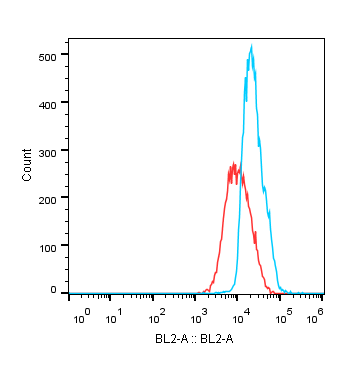


C


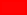


Nile Red

Nile Red + extract 200 µM


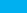

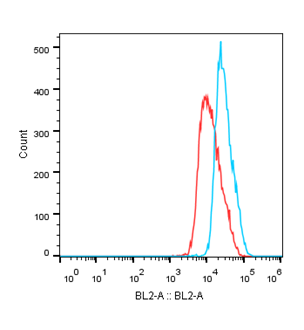


B


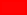


Nile Red

Nile Red + extract 200 µM


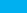

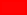


Nile Red

Nile Red + extract 200 µM


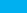

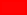


Nile Red

Nile Red + extract 200 µM


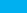

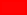


Nile Red

Nile Red + extract 50 µM


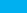

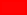


Nile Red

Nile Red + extract 12.5 µM


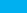

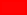


Nile Red

Nile Red + extract 12.5 µM


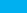

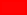


Nile Red

Nile Red + extract 25 µM


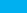


**Fig. S1**. Flow cytometric determinations of the effect of *Acalypha communis* extract on Nile Red accumulation in: (A) *Candida albicans* 1114; (B) *Saccharomyces cerevisia*e AD/CaMDR1; (C) *Saccharomyces cerevisia*e AD/CaCDR1; and (D) *Candida glabrata* 109.

Nile Red Fluorescence

Nile Red Fluorescence

Nile Red Fluorescence


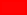


Nile Red

Nile Red + extract 25 µM


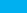

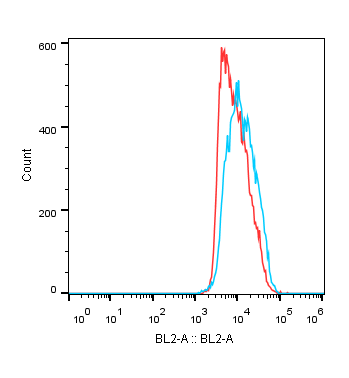

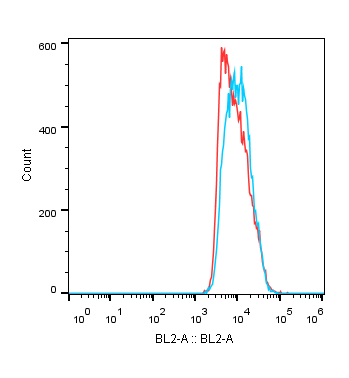

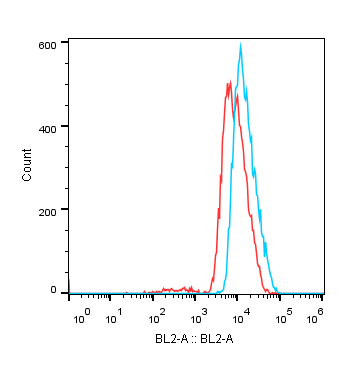

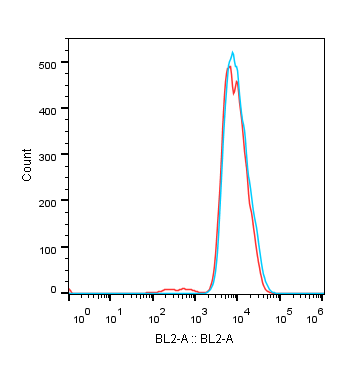

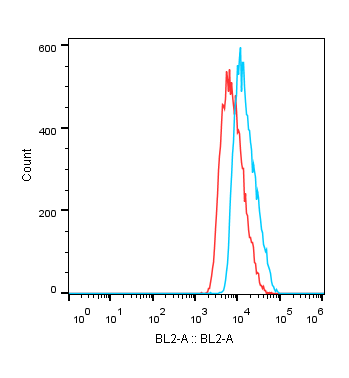

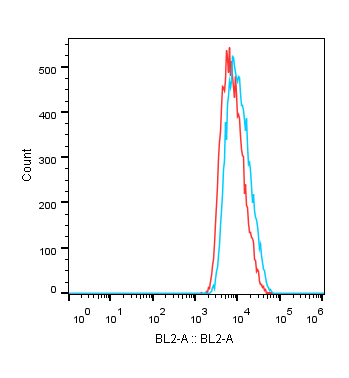


Nile Red Fluorescence


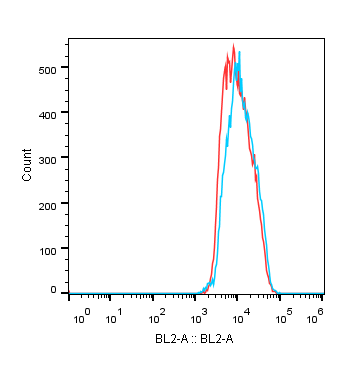

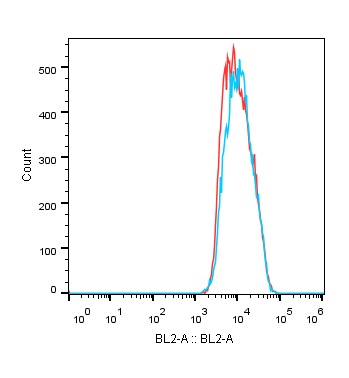

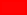


Nile Red

Nile Red + extract 50 µM


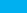

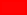


Nile Red

Nile Red + extract 50 µM


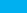

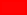


Nile Red

Nile Red + extract 50 µM


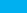


**Fig. S2**

A

B

C

D


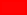


Nile Red

Nile Red + extract 200 µM


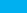

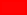


Nile Red

Nile Red + extract 200 µM


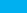

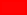


Nile Red

Nile Red + extract 200 µM


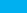

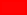


Nile Red

Nile Red + extract 200 µM


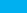


**Fig. S2.** Flow cytometric determinations of the effect of *Solanum atriplicifolium* extract on Nile Red accumulation in: (A) *Candida albicans* 1114; (B) *Saccharomyces cerevisiae* AD/CaMDR1; (C) *Saccharomyces cerevisiae* AD/CaCDR1; and (D) *Candida glabrata* 109.


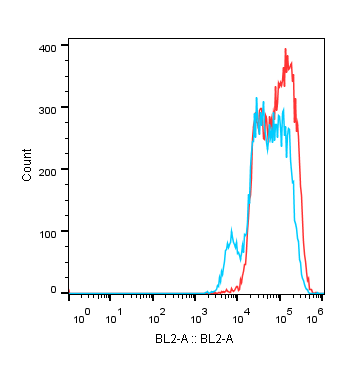


A


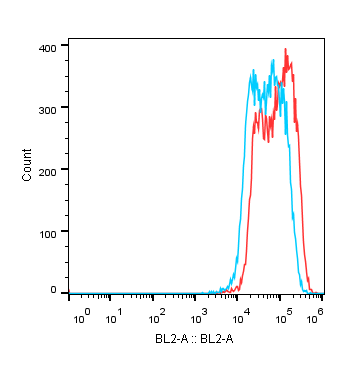


B


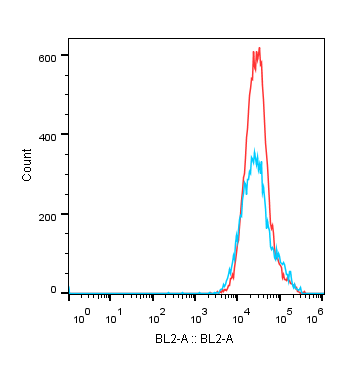

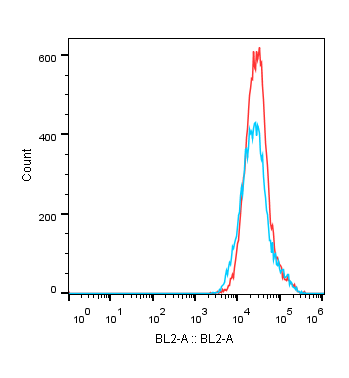


*Saccharomyces cerevisiae* AD1-8u^-^

*Candida albicans* ATCC 90028


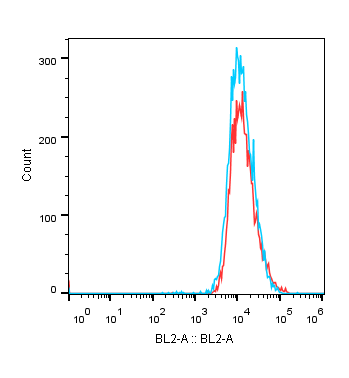

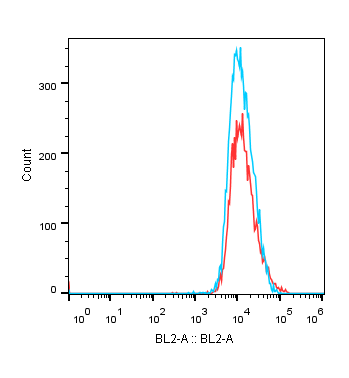


**Fig. S3**

*Candida glabrata* ATCC 2001

**Fig. S3.** Determination by flow cytometry of the effect of (A) *Acalypha communis* and (B) *Solanum atriplicifolium* extracts on *Saccharomyces cerevisiae* AD1-8u^-^, *Candida albicans* ATCC 90028 and *Candida glabrata* ATCC 2001. Histograms represent cells treated with Nile Red alone in red and cells treated with Nile Red and extracts in blue.
